# Nutrient enrichment, propagule pressure, and herbivory interactively influence the competitive ability of an invasive alien macrophyte *Myriophyllum aquaticum*

**DOI:** 10.3389/fpls.2024.1411767

**Published:** 2024-05-30

**Authors:** Ru Huang, Ayub M. O. Oduor, Yimin Yan, Weicheng Yu, Chuanxin Chao, Lei Dong, Shaofei Jin, Feng Li

**Affiliations:** ^1^ Key Laboratory of Agro-Ecological Processes in Subtropical Region, Institute of Subtropical Agriculture, Chinese Academy of Sciences, Changsha, Hunan, China; ^2^ Dongting Lake Station for Wetland Ecosystem Research, Institute of Subtropical Agriculture, Chinese Academy of Sciences, Changsha, Hunan, China; ^3^ National Field Scientific Observation and Research Station of Dongting Lake Wetland Ecosystem in Hunan Province, Institute of Subtropical Agriculture, Chinese Academy of Sciences, Changsha, Hunan, China; ^4^ University of Chinese Academy of Sciences, Chinese Academy of Sciences, Beijing, China; ^5^ Department of Applied Biology, Technical University of Kenya, Nairobi, Kenya; ^6^ Department of Geography and Oceanography, Minjiang University, Fuzhou, China; ^7^ Technology Innovation Center for Ecological Conservation and Restoration in Dongting Lake Basin, Ministry of Natural Resources, Changsha, Hunan, China

**Keywords:** biological invasions, eutrophication, macrophytes, nutrient availability, propagule pressure, native herbivore

## Abstract

**Introduction:**

Freshwater ecosystems are susceptible to invasion by alien macrophytes due to their connectivity and various plant dispersal vectors. These ecosystems often experience anthropogenic nutrient enrichment, favouring invasive species that efficiently exploit these resources. Propagule pressure (reflecting the quantity of introduced individuals) and habitat invasibility are key determinants of invasion success. Moreover, the enemy release hypothesis predicts that escape from natural enemies, such as herbivores, allows alien species to invest more resources to growth and reproduction rather than defense, enhancing their invasive potential. Yet, the combined impact of propagule pressure, herbivory, and nutrient enrichment on the competitive dynamics between invasive alien macrophytes and native macrophyte communities is not well understood due to a paucity of studies.

**Methods:**

We conducted a full factorial mesocosm experiment to explore the individual and combined effects of herbivory, nutrient levels, propagule pressure, and competition on the invasion success of the alien macrophyte *Myriophyllum aquaticum* into a native macrophyte community comprising *Vallisneria natans, Hydrilla verticillata*, and *Myriophyllum spicatum*. This setup included varying *M. aquaticum* densities (low vs. high, simulating low and high propagule pressures), two levels of herbivory by the native snail *Lymnaea stagnalis* (herbivory vs no-herbivory), and two nutrient conditions (low vs. high). *Myriophyllum aquaticum* was also grown separately at both densities without competition from native macrophytes.

**Results:**

The invasive alien macrophyte *M. aquaticum* produced the highest shoot and total biomass when simultaneously subjected to conditions of high-density intraspecific competition, no herbivory, and low-nutrient availability treatments. Moreover, a high propagule pressure of *M. aquaticum* significantly reduced the growth of the native macrophyte community in nutrient-rich conditions, but this effect was not observed in nutrient-poor conditions.

**Discussion:**

These findings indicate that *M. aquaticum* has adaptive traits enabling it to flourish in the absence of herbivory (supporting the enemy release hypothesis) and in challenging environments such as intense intraspecific competition and low nutrient availability. Additionally, the findings demonstrate that when present in large numbers, *M. aquaticum* can significantly inhibit the growth of native macrophyte communities, particularly in nutrient-rich environments. Consequently, reducing the propagule pressure of *M. aquaticum* could help control its spread and mitigate its ecological impact. Overall, these findings emphasize that the growth and impacts of invasive alien plants can vary across different habitat conditions and is shaped by the interplay of biotic and abiotic factors.

## Introduction

A significant proportion of the alien (i.e., non-native) plant species that have been introduced to new geographical regions globally through human activities have become invasive whereby they proliferate and significantly impact native biodiversity and ecosystem functioning (Richardson et al., 2000; Simberloff et al., 2013; Hautier et al., 2018; Blackburn et al., 2019). Consequently, gaining insights into the ecological processes that facilitate invasions is an important objective in the field of ecology. Two critical factors that have been identified to facilitate the invasions of alien plants are the release from natural enemies (Keane and Crawley, 2002) and nutrient enrichment (Dukes et al., 1999). The enemy release hypothesis is a critical concept in understanding the dynamics of biological invasions and the success of alien species in new environments (Keane and Crawley, 2002). It posits that when species are introduced to new areas, they often escape the control of their natural enemies, including herbivores, which limit their population in their native range (Keane and Crawley, 2002). The release from specialized natural enemies should allow introduced species to allocate resources that would have been used for defense mechanisms towards greater growth and reproduction, leading to increased invasion success (Keane and Crawley, 2002). The enemy release hypothesis is supported by empirical evidence showing that invasive alien plant species tend to interact with fewer species of herbivores and exhibit lower levels of defense mechanisms but greater growth and reproductive capacities in their introduced ranges compared to their native ranges (Colautti et al., 2004; Liu and Stiling, 2006; Meijer et al., 2016; Oduor, 2022). Nutrient enrichment refers to the increase in nutrient availability in ecosystems, often as a result of human activities such as agriculture, wastewater discharge, and the deposition of atmospheric nitrogen (Bobbink et al., 1998; Maharjan et al., 2022). This enrichment can favour invasive species over co-occurring native plant species due to: 1) inherently higher resource-use efficiency in invasive plants compared to native species, allowing invaders to capitalize on increased nutrient availability more effectively (Funk and Vitousek, 2007); 2) inherently faster growth rates of invasive plants (van Kleunen et al., 2010) and which can be enhanced by nutrient enrichment (Liu et al., 2017), allowing the invaders to quickly dominate a landscape, outcompeting slower-growing native species; and 3) altered competition dynamics whereby nutrient enrichment shifts competition dynamics in favour of invasive species by enhancing their competitive abilities (Abonyo and Oduor, 2024) or by disrupting the nutrient-use strategies of native species (Liu et al., 2016; Tan et al., 2019).

The invasion success of alien plant species is often a result of complex interactions between biotic and abiotic factors (Gioria et al., 2023). However, the effects of release from natural enemies and nutrient enrichment on plant invasions have often been examined in isolation rather than in conjunction, particularly in aquatic ecosystems (Yan et al., 2024). Examining these factors separately can provide insights into their individual roles in facilitating invasions, but it also presents a limitation by not fully accounting for the interactions between biotic and abiotic factors that can influence invasion success. For instance, nutrient enrichment may favour invasive plants over natives, but the real advantage might only become pronounced when combined with the release from natural enemies. Conversely, in nutrient-poor environments, the impact of enemy release might be less pronounced because all plants, including invaders, are limited by the availability of nutrients (Colautti et al., 2004). Freshwater ecosystems, which include rivers, lakes, streams, and wetlands, are particularly susceptible to invasions due to their interconnected nature and the various vectors for plant dispersal they offer, such as water flow, animal movement, and the frequent human activities that occur in and around them, such as shipping, fishing, and recreational use, which often introduce alien plant species (Havel et al., 2015; Kernan, 2015; Tamayo and Olden, 2017). These ecosystems also frequently experience nutrient enrichment from runoffs containing fertilizers and other pollutants, creating ripe conditions for invasive species that can exploit these resources more efficiently than native species (Teixeira et al., 2017). But studies that address how nutrient enrichment and herbivory may interact to influence the success of invasive and native macrophyte species in the freshwater ecosystems remain rare (Yan et al., 2024).

Every invasion must commence with the introduction of viable propagules, with invasion success hypothesized to scale proportionally with the propagule pressure and the degree of invasibility of the habitat (Lockwood et al., 2005; Simberloff et al., 2013). Propagule pressure refers to the number and frequency of reproductive structures of a plant introduced into a system (Lockwood et al., 2005; Simberloff et al., 2013). High propagule pressure is thought to increase the likelihood of successful invasions, as species with high propagule pressure may overcome limited genetic variation, demographic stochasticity, biotic resistance from native species, and abiotic factors such as unsuitable climate or nutrient availability (Colautti et al., 2006; Simberloff, 2009). Despite the recognized importance of propagule pressure in invasion biology, our understanding of the role of propagule pressure and its interactions with biotic and abiotic components of the environment is limited for most invasive plant species (Barney et al., 2016), particularly in freshwater ecosystems (Thomaz, 2023).

China is home to over 110,000 lakes, covering 0.8% of the country’s total land area (Liu and Qiu, 2007). More than 2,300 of these lakes each covers an area greater than 1 square kilometer (Liu and Qiu, 2007). Approximately one-third of all lakes in China are freshwater bodies (Qin et al., 2013). Most of China’s lakes have experienced excessive nutrient input, primarily nitrogen and phosphorus, due to anthropogenic activities (Jin, 2003; Qin et al., 2013). The levels of nutrient enrichment vary across different regions (Huo et al., 2013). This nutrient enrichment is a major ecological and environmental issue, linked to the extinction of submerged plants and a decline in biodiversity (Qin et al., 2013). In addition to nutrient enrichment, several freshwater lakes in China have faced invasions by several non-native macrophytes (Zhan et al., 2017), including *Myriophyllum aquaticum* (Yan et al., 2024). However, despite the prevalence of nutrient enrichment and alien macrophyte invasions in aquatic ecosystems, the combined effects of these two factors on native macrophyte communities remain poorly understood due to a lack of comprehensive research in this area (Yan et al., 2024).

In this study, we conducted a full factorial mesocosm experiment to examine the main and potential interactive effects of herbivory, nutrient availability, propagule pressure, and competition on the invasion success of the alien macrophyte *M. aquaticum* into a native macrophyte community. We assembled a native macrophyte community of three species, including *Vallisneria natans*, *Hydrilla verticillate* and *Myriophyllum spicatum*, which we then invaded with *M. aquaticum* at low density (one individual; representing low-propagule pressure) vs high density (four individuals; representing high-propagule pressure) and fully crossed with two levels of herbivory by a native snail *Lymnaea stagnalis* (herbivory vs no-herbivory) and two levels of nutrient availability (low-nutrient vs high-nutrient). We also grew *M. aquaticum* separately at low density and high density in the absence of competition from the native macrophyte community. We hypothesized that: (1) *M. aquaticum* would have a significantly stronger competitive effect on the biomass of native macrophyte community when the invader is introduced at high density; (2) nutrient enrichment would enhance competitive effect of *M. aquaticum* on the native macrophyte community, especially in the presence of herbivory pressure on the native macrophyte community.

## Materials and methods

### Study system

We carried out the mesocosm experiment at the Dongting Lake Wetland Ecosystem Observation and Research Station of the Chinese Academy of Sciences, Yueyang, Hunan province (29.30°N, 150.74°E) using the invasive alien macrophyte *M. aquaticum* (Haloragidaceae) and co-occurring native macrophytes *V. natans* (Hydrocharitaceae), *H. verticillate* (Hydrocharitaceae) and *M. spicatum* (Haloragaceae). *Myriophyllum aquaticum* occurs in stagnant waters (Orchard, 1981). The species reproduces vegetatively, and hence any events that can cause fragmentation will facilitate its spread and growth (Hough et al., 1991). The three native macrophyte species *V. natans*, *H. verticillate* and *M. spicatum* occur in submerged water and can reproduce vegetatively. While *H. verticillate* and *V. natans* can both grow in eutrophic and oligotrophic lakes, *M. spicatum* is most commonly found in eutrophic lakes and is more likely to flourish under high nutrient conditions (Aiken et al., 1979). As a herbivore, we used the native pond snail *L. stagnalis*, which is commonly abundant in lentic systems in Asia (Zhang et al., 2018). It occurs in littoral areas and is an omnivorous species feeding mainly on algae (Brönmark, 1989), aquatic plants (Lance et al., 2006), insects, snails, and fish (Elger et al., 2004).

### Experimental set-up

On July 12, 2020, we purchased seedlings of the four macrophyte species from a local company producing seedlings (Guangzhou Beishanshui Ecological Technology Co., Ltd, Ezhou, China), and then kept them in the pond water. On July 19, 2020, we prepared 112 mesocosms that comprised of pails that each measured 70 cm in height, 28 cm at the top diameter, and 26 cm at the bottom diameter. We wrapped the pails with tinfoil to prevent overheating caused by the sun rays. We filled each pail to 10 cm with soil that we collected from the research station. To avoid potential interferences caused by the presence of macrophyte propagules in the lake sediment, we opted to use terrestrial soil as the substrate for the experiment instead of using soil collected from the lake. We sieved the soil to remove large stones and debris. After that, we added groundwater into each mesocosm to a depth of 20 cm.

**Figure 1:**
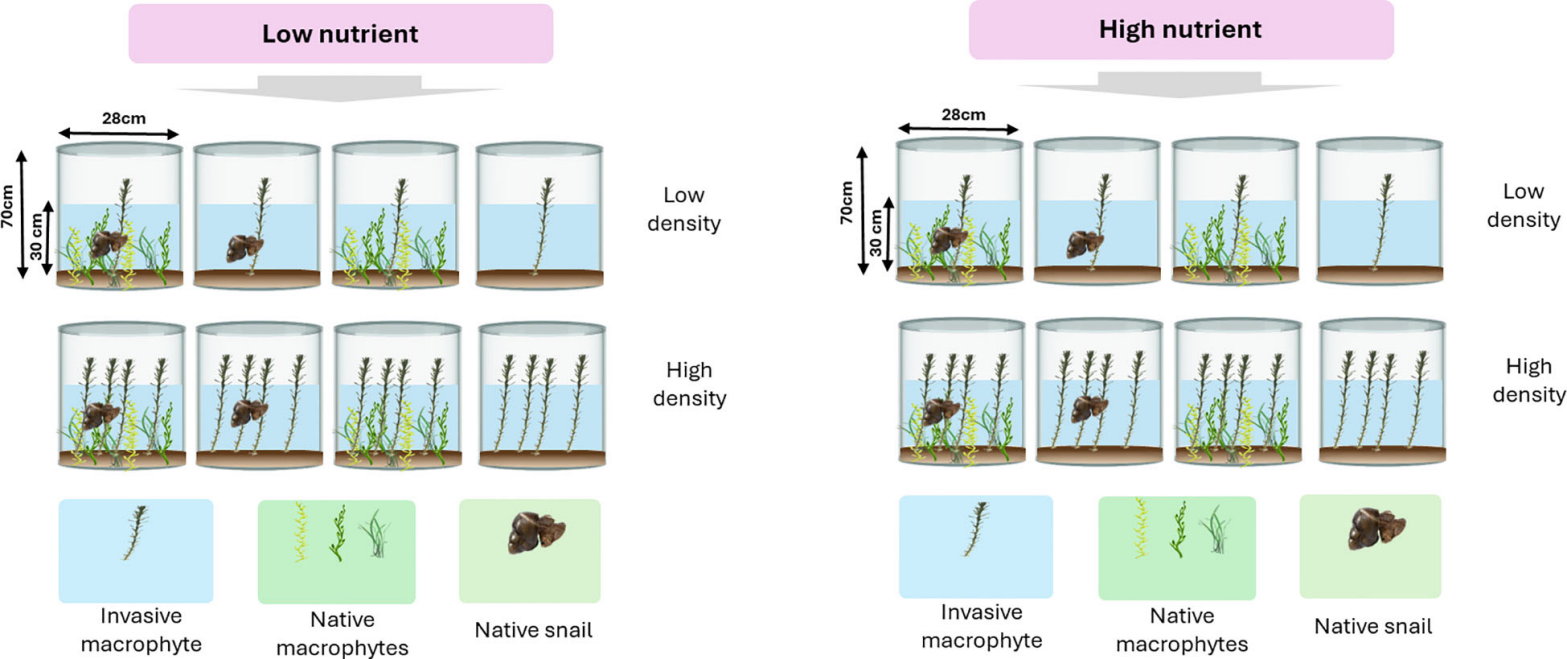
A schematic of experimental design. We created a native macrophyte community of three species, including *Vallisneria natans*, *Hydrilla verticillate* and *Myriophyllum spicatum*, which we then invaded with *M. aquaticum* at low density (one individual; representing low-propagule pressure) vs high density (four individuals; representing high-propagule pressure) and fully crossed with two levels of herbivory by a native snail *Lymnaea stagnalis* (herbivory vs. no-herbivory) and two levels of nutrient availability (low-nutrient vs high-nutrient). We also grew *M. aquaticum* separately at low density and high density in the absence of competition from the native macrophyte community.

The experimental setup included varying *M. aquaticum* densities (low vs. high, simulating low and high propagule pressures), two levels of herbivory by the native snail *L. stagnalis* (herbivory vs no-herbivory), and two nutrient conditions (low-nutrient vs high-nutrient). *Myriophyllum aquaticum* was also grown separately at both densities without competition from native macrophytes. Each treatment combination was replicated seven times, which resulted in a total of 112 mesocosms (**Figure 1**). We created a native macrophyte community in each pail by transplanting similar-sized stem cuttings of *H. verticillate* and *M. spicatum* and seedlings of *V. natans*. Then, we transplanted the alien macrophyte *M. aquaticum* at low and high-propagule pressure into the center of a half of the pails (n=56) containing the native macrophyte community. In the other half of the pails (n=56), we grew *M. aquaticum* separately at low and high-propagule pressure in the absence of competition from the native macrophytes. For low-level propagule pressure, we transplanted one individual *M. aquaticum* and for high-level propagule pressure, we transplanted four individuals of *M. aquaticum* into the individual pails. Immediately thereafter, we covered each mesocosm with an insect net to avoid ingress of insects from the ambient environment. We replaced dead seedlings or stem cuttings within four days of transplant. On August 12, 2020, we added water to each mesocosm to a depth of 30 cm, and then applied the nutrient treatment once using a soluble fertilizer. We applied the fertilizer at the concentration of 0.2 mg/L and 1 mg/L for low-nutrient and high-nutrient treatments, respectively. The nutrient concentrations that we applied mimic those that have been observed in Lake Dongting, and have been shown to have different effects on macrophyte growth (Yan et al., 2024). The nutrient content of the fertilizer was as follows: in the low-nutrient treatment, 0.36 g of the fertilizer contained 0.072 g of total nitrogen and 0.072 g of available phosphate, while in the high-nutrient treatment, 1.44 g of the fertilizer contained 0.288 g of total nitrogen and 0.288 g of available phosphate. On August 14, 2020, we collected *L. stagnalis* individuals from pounds nearby and kept them in pond water with similar conditions as those in the mesocosm for a week to acclimatize. We then introduced two similar-sized individuals of the herbivore (each measuring 2-2.5 cm in length) into each of the pails that were designated to receive herbivore treatment. On October 2, 2020, we removed all the snails and harvested root and shoot biomass of all the plants per pail separately. All plant samples were dried at 65°C for 3 days to constant weight and then weighed. The dry biomass was then subjected to statistical analyses as detailed below.

### Statistical analysis

To test the individual and combined effects of nutrient availability, propagule pressure, herbivory by the native snail, and competition on the growth performance of the invasive alien macrophyte *M. aquaticum* and the native macrophyte communities, we fitted general linear models. In the models, we included total biomass, shoot biomass, root biomass, root mass fraction (i.e., root biomass/total biomass per individual *M. aquaticum*), and proportional shoot biomass (i.e., shoot biomass per individual *M. aquaticum*/total shoot biomass per pail) of the invasive alien macrophyte as well as shoot biomass and total biomass of the native macrophyte community as the dependent variables. We treated propagule pressure (low-propagule pressure [low-density] vs high-propagule pressure [high density]), nutrient availability (low-nutrient vs high-nutrient), herbivory by the native snail (herbivory vs no-herbivory), competition by the native community (competition vs no-competition) and all possible four-way, three-way, and two-way interactions among them as independent variables. The values of the dependent variables were square-root-transformed to attain normality of residuals. We conducted all the analyses in R 4.1.0 (R Core Team, 2020).

## Results

### Growth responses of the invasive alien macrophyte *M. aquaticum*

**Figure 2:**
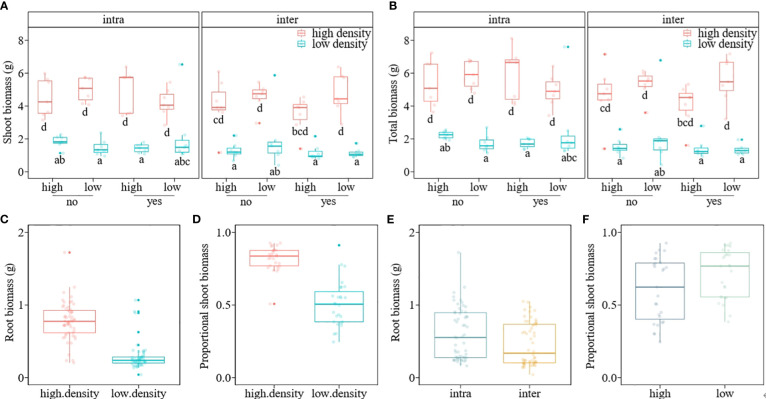
Boxplots showing the values of shoot biomass **(A)**, total biomass **(B)**, root biomass **(C, E)** and proportional shoot biomass **(D, F)** of an invasive alien macrophyte *Myriophyllum aquaticum* under different levels of herbivory (herbivory [yes] vs. no-herbivory [no]), propagule pressure (low density vs. high density), nutrient (low vs. high), and competition (intraspecific [intra] vs. interspecific from a native macrophyte community [inter]) treatments. Boxes show the interquartile range around the median, whiskers extend to 1.5× the interquartile range (or to the minimum or maximum observed value within that). Data points beyond the whiskers are outliers (marked by circles).

**Figure 3:**
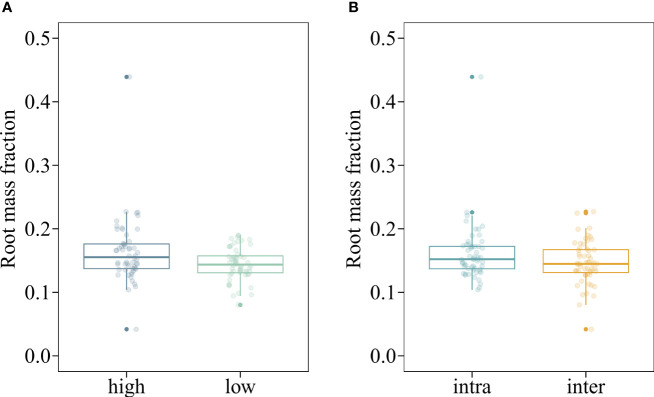
Boxplots showing the values of root mass fraction of an invasive alien macrophyte *Myriophyllum aquaticum* under different levels of nutrient (low vs. high) **(A)** and competition (intraspecific [intra] vs. interspecific from a native macrophyte community [inter]) **(B)** treatments. Boxes show the interquartile range around the median, whiskers extend to 1.5× the interquartile range (or to the minimum or maximum observed value within that). Data points beyond the whiskers are outliers (marked by circles).

The mean shoot biomass and total biomass of the invasive alien macrophyte *M. aquaticum* were significantly influenced by four-way interaction among herbivory, nutrient availability, propagule pressure, and competition (**Table 1**). Specifically, *M. aquaticum* produced the greatest mean shoot biomass and total biomass when simultaneously grown under intraspecific competition at high-density and in the absence of herbivory and under low-nutrient treatment (**Figures 2A, B**). In contrast, *M. aquaticum* produced the least amount of shoot biomass and total biomass when simultaneously grown under interspecific competition, low-nutrient, low-density and in the presence of herbivory (**Figures 2A, B**). Shoot biomass and total biomass of the invasive macrophyte species were also significantly influenced by the main effects of propagule pressure and competition (**Table 1**). Root biomass of *M. aquaticum* was significantly influenced by propagule pressure and competition (**Table 1**). Specifically, *M. aquaticum* produced significantly higher root biomass (178.57%) when grown at high density than at low density (**Figure 2C**). Additionally, *M. aquaticum* produced higher root biomass (30.43%) in intraspecific competition compared to interspecific competition (**Figure 2E**). Root mass fraction was significantly influenced by the separate effects of nutrient and competition treatments (**Table 1**). Specifically, root mass fraction was significantly higher under high (13.19%) than low nutrient treatments (**Figure 3A**). Moreover, root mass fraction was significantly higher under intraspecific (10.27%) than interspecific competition (**Figure 3B**). Proportional shoot biomass of the invasive alien macrophyte was significantly influenced by the separate effects of nutrient and propagule pressure treatments (**Table 2**). Specifically, the mean proportional shoot biomass was significantly lower under high (-15.95%) than low nutrient treatment (**Figure 2F**). The proportional shoot biomass was also significantly higher at high (61.35%) than low density (**Figure 2D**).

**Table 1:** Results of a general linear model that tested the main and interactive effects of herbivory (herbivory vs. no-herbivory), nutrient enrichment (low vs. high), propagule pressure of an invasive alien macrophyte *Myriophyllum aquaticum* (low vs. high density) and competition (intraspecific vs interspecific from a native macrophyte community) on shoot biomass, root biomass, total biomass, and root mass fraction of the invader.

Factor	Shoot biomass		Root biomass		Total biomass		Root mass fraction	
	F	*P*	F	*P*	F	*P*	F	*P*
Herbivory	1.03	0.313	0.028	0.867	0.87	0.352	1.75	0.188
Nutrient	2.34	0.129	0.010	0.921	1.62	0.207	4.40	**0.038**
Propagule pressure	239.95	**<0.001**	177.39	**<0.001**	247.29	**<0.001**	0.74	0.393
Competition	5.18	**0.025**	13.37	**<0.001**	6.82	**0.010**	4.25	**0.042**
Herbivory × Nutrient	0.02	0.887	0.16	0.689	0.007	0.934	0.54	0.466
Herbivory × Propagule pressure	0.02	0.894	0.015	0.904	0.023	0.880	0.26	0.613
Nutrient × Propagule pressure	0.25	0.616	1.48	0.225	0.43	0.513	3.06	0.083
Herbivory × Competition	0.44	0.507	0.02	0.893	0.36	0.552	0.53	0.469
Nutrient × Competition	2.02	0.158	3.20	0.077	2.46	0.120	0.01	0.925
Propagule pressure × Competition	0.07	0.794	0.57	0.449	0.012	0.898	1.00	0.320
Herbivory × Nutrient × Propagule pressure	0.37	0.546	1.04	0.309	0.49	0.484	0.34	0.557
Herbivory × Nutrient × Competition	0.03	0.858	0.01	0.921	0.03	0.855	0.11	0.738
Herbivory × Propagule pressure × Competition	0.59	0.443	0.07	0.795	0.46	0.501	0.34	0.563
Nutrient × Propagule pressure × Competition	0.75	0.388	2.11	0.149	0.95	0.332	1.57	0.213
Herbivory × Nutrient × Propagule pressure × Competition	4.67	**0.033**	3.32	0.071	5.03	**0.027**	0.56	0.456

Significant factors (*P*<0.05) are highlighted in bold font text, while marginally significant factors are underlined.

**Table 2:** Results of a general linear model that tested the main and interactive effects of herbivory (herbivory vs. no-herbivory), nutrient enrichment (low vs. high), and propagule pressure of an invasive alien macrophyte *Myriophyllum aquaticum* (low vs. high density) on proportional shoot biomass of the invader.

Factor	Proportional shoot biomass	
	F	*P*
Herbivory	0.11	0.740
Nutrient	13.16	**0.001**
Propagule pressure	92.73	**<0.001**
Herbivory × Nutrient	0.73	0.398
Herbivory × Propagule pressure	0.33	0.571
Nutrient × Propagule pressure	2.16	0.148
Herbivory × Nutrient × Propagule pressure	0.92	0.342

Significant factors (*P*<0.05) are highlighted in bold font text.

### Growth responses of native macrophyte community

**Figure 4:**
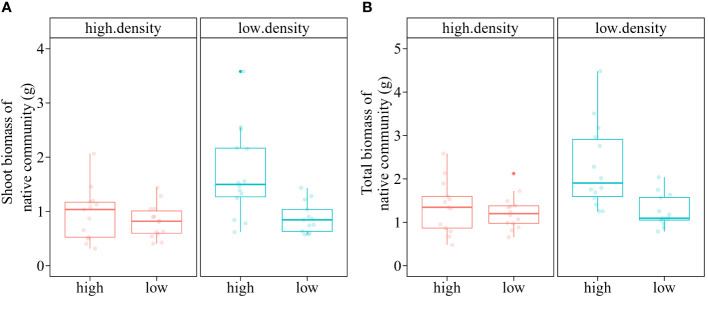
Boxplots showing the values of shoot biomass **(A)** and total biomass **(B)** of a native macrophyte community that was invaded by an invasive alien macrophyte *Myriophyllum aquaticum* at low vs high propagule pressures (i.e., low density vs. high density) under low vs. high nutrient treatments. Boxes show the interquartile range around the median, whiskers extend to 1.5× the interquartile range (or to the minimum or maximum observed value within that). Data points beyond the whiskers are outliers (marked by circles).

The total biomass and shoot biomass of the native macrophyte community were significantly influenced by the main and interactive effects of nutrient treatment and propagule pressure of the alien macrophyte *M. aquaticum* (**Table 3**). Specifically, when grown in high nutrient conditions, the native macrophyte community produced significantly less shoot biomass (-43.78%) when heavily invaded by *M. aquaticum* (i.e., high propagule pressure of *M. aquaticum*), compared to when only lightly invaded by *M. aquaticum* (i.e., low propagule pressure of *M. aquaticum*) (**Figure 4A**). In contrast, when grown in low nutrient conditions, the native macrophyte community produced similar mean amounts of shoot biomass when heavily invaded by *M. aquaticum* (0.82 g) as when only lightly invaded by *M. aquaticum* (0.88 g) (**Figure 4A**). A similar pattern was observed for the total biomass (**Figure 4B**). Specifically, when grown in high nutrient conditions, the native macrophyte community produced significantly less total biomass (-68.14%) when heavily invaded by *M. aquaticum*, compared to when only lightly invaded by *M. aquaticum* (**Figure 4B**). However, under low-nutrient growth condition, the native macrophyte community produced similar mean amounts of total biomass when heavily invaded by *M. aquaticum* (1.23 g) as when lightly invaded by *M. aquaticum* (1.26 g) (**Figure 4B**).

**Table 3:** Results of a general linear model that tested the main and interactive effects of herbivory (herbivory vs. no-herbivory), nutrient enrichment (low vs. high), and propagule pressure of an invasive alien macrophyte *Myriophyllum aquaticum* (low vs. high density) on total biomass and shoot biomass of a native macrophyte community.

Factor	Total biomass		Shoot biomass	
	F	*P*	F	*P*
Herbivory	1.21	0.277	0.71	0.404
Nutrient	10.82	**0.002**	10.42	**0.002**
Propagule pressure	8.26	**0.006**	8.35	**0.006 **
Herbivory × Nutrient	1.03	0.315	1.30	0.260
Herbivory × Propagule pressure	0.34	0.562	0.02	0.882
Nutrient × Propagule pressure	7.05	**0.011**	5.30	**0.026**
Herbivory × Nutrient × Propagule pressure	2.01	0.163	0.93	0.341

Significant factors (*P*<0.05) are highlighted in bold font text.

## Discussion

Our multi-factor mesocosm experiment revealed that the invasive alien macrophyte *M. aquaticum* exhibited optimal growth under simultaneous conditions of high-density intraspecific competition, absence of herbivory, and low nutrient availability (**Figures 2A, B**). Moreover, high propagule pressure of *M. aquaticum* significantly impaired the growth of a native macrophyte community in nutrient-rich conditions, although this effect was not seen in nutrient-poor conditions (**Figures 4A, B**). These findings indicate that *M. aquaticum* has adaptive traits enabling it to flourish without herbivores (supporting the enemy release hypothesis) and in challenging environments, such as intense intraspecific competition and low nutrient availability. Additionally, the findings demonstrate that when present in large numbers, *M. aquaticum* can significantly inhibit the growth of native macrophyte communities, particularly in nutrient-rich environments. Therefore, reducing the propagule pressure of *M. aquaticum* could help control its spread and mitigate its ecological impact. Overall, these findings emphasize that the growth and impact of invasive alien plants can differ across various habitats and are influenced by a combination of biotic and abiotic factors (Theoharides and Dukes, 2007).

Our results showing that the invasive alien macrophyte *M. aquaticum* attained the highest mean shoot and total biomass under conditions of high-density intraspecific competition, no herbivory, and low nutrient availability (**Figures 2A, B**) indicates that *M. aquaticum* is well-adapted to thrive under these specific environmental conditions. This pattern could be attributed to several mechanisms. Firstly, through positive feedback loops, invasive plants growing at high-density can change their environment in ways that promote their own growth, such as by altering the soil or water chemistry (Gaertner et al., 2014). Secondly, at high-densities, invasive species may more effectively utilize resources, including nutrients, light, and space, leveraging their typically higher resource-use efficiency (Antonovics and Levin, 1980; Čuda et al., 2015). Third, intraspecific facilitation among individuals may have enhanced growth of *M. aquaticum* at high density. In fact, some studies suggest that individuals of the same invasive species can facilitate each other’s growth through processes such as alteration of microhabitat conditions or through mutualistic relationships with other organisms, which becomes more pronounced at higher densities (Zhang and Tielbörger, 2019). Fourth, the absence of herbivory pressure likely allowed *M. aquaticum* to achieve its highest biomass. This suggests that herbivory can be a significant limiting factor for the growth and spread of this invasive macrophyte. When herbivores are present, they may consume parts of the plant, reducing its overall biomass and potentially limiting its competitive ability. These findings contribute to the broader understanding of invasive species dynamics, particularly how high propagule pressure and escape from herbivory can advantage invasive species in nutrient-limited environments.

The finding that the alien macrophyte *M. aquaticum* produced significantly higher root biomass at higher density than low density independent of nutrient level (**Figure 2C**), suggests that *M. aquaticum* invests more in root system development when individuals are closely packed. Plants have long been observed to allocate more biomass to the organs that enhance the acquisition of the most limited resource, a phenomenon known as ‘optimal partitioning’ (Gedroc et al., 1996; Shipley and Meziane, 2002; Mokany et al., 2006). Consequently, the relative distribution of vegetative biomass to roots (root mass fraction), stems (stem mass fraction), and leaves (leaf mass fraction) exhibits predictable responses to most abiotic stresses (Poorter et al., 2012). Based on the optimal partitioning theory, it would be expected that *M. aquaticum* would produce more root biomass under low-nutrient conditions compared to high-nutrient conditions. However, the findings of this study do not align with this expectation. In support of the present results, other studies have shown that plants can increase their biomass allocation to the roots when grown at high density independent of nutrient level (Rehling et al., 2021). By developing larger root systems at high density, *M. aquaticum* may exclude native plants and other competitors from accessing vital resources, such as nutrients. Moreover, a larger root system in *M. aquaticum* at high density may support more vigorous shoot growth and potentially enhance the invader’s reproductive output, further aiding its invasive spread. This proposition is supported by our results showing that *M. aquaticum* produced significantly higher proportional shoot biomass at high density than at low density (**Figure 2D**).

The observation that the invader *M. aquaticum* developed a larger root system (**Figure 2E**) and allocated a higher proportion of its total biomass to the roots (**Figure 3B**) when grown in intraspecific competition, compared to interspecific competition, indicates that the invader experienced more intense intraspecific competition for resources, such as nutrients. This could be because all the individuals have similar resource requirements and growth strategies, leading to more intense competition when growing in monospecific stands. Broadly, these results align with a prediction of the classical theory on plant coexistence, which states that intraspecific competition should have stronger suppressive effects on growth and fecundity of focal plants than interspecific competition because individuals of the same species share similar resource requirements (Aarssen, 1983; Chesson, 2000). When the effects of intraspecific competition are stronger than those of interspecific competition, each species in a community should limit its own population growth more than it limits the population growth of its competitors (Adler et al., 2018). This should result in a high mortality rate at high densities (also known as the self-thinning rule; Westoby, 1984). Research investigating how intraspecific competition influences biomass allocation has yielded inconsistent findings. Some studies report that as density increases, plants allocate more biomass to their roots, increasing the root mass fraction (Lentz, 1999; Berendse and Möller, 2009). Other studies found that higher density leads to increased biomass allocation to leaves (leaf mass fraction) or stems (stem mass fraction) (Meekins and McCarthy, 2000; Forster et al., 2011; Hecht et al., 2016). Yet other studies did not observe any changes in biomass allocation patterns due to varying densities (Flint and Patterson, 1983). The varying results from studies examining the effects of density on biomass allocation patterns could potentially be attributed to differences in the characteristics of the plant species (e.g., life cycle and functional group) under investigation and the environmental conditions under which the studies are performed (Rehling et al., 2021).

The proportional shoot biomass of *M. aquaticum* was significantly higher under low-nutrient than high-nutrient treatment (**Figure 2F**), which could be an adaptive strategy by the macrophyte to maximize light capture and photosynthesis, thereby compensating for the limited nutrient availability. Although invasive plant species are primarily found in disturbed environments with abundant resources, many species have successfully invaded ecosystems characterized by low nutrient, water, and light availability (Funk and Vitousek, 2007; Funk, 2013). Plant species adapted to these low-resource systems often exhibit traits associated with resource conservation, such as efficient use of available resources (Funk and Vitousek, 2007). Future studies may explore the physiological mechanisms that underlie adaptation of *M. aquaticum* to a gradient of nutrient levels.

The root mass fraction of *M. aquaticum* was significantly higher under high-nutrient than low-nutrient treatment (**Figure 3A**), which contrasts with the theoretical prediction that plants should reduce biomass allocation to the roots when growing in nutrient-rich media (Gedroc et al., 1996; Shipley and Meziane, 2002; Mokany et al., 2006). The increased allocation of biomass to roots in high-nutrient environments suggests that *M. aquaticum* may enhance its ability to absorb and process these nutrients efficiently. A larger root system can exploit the nutrient-rich environment more effectively, which in turn supports higher relative growth rate and competitive ability. Plant species from highly productive habitats usually have higher relative growth rates (Poorter and Remkes, 1990) and are more competitive in nutrient-rich habitats than species from nutrient-poor habitats (Klinerová and Dostál, 2020). Invasive plant species often occur in nutrient-rich habitats (Chytrý et al., 2008). Thus, nutrient enrichment may enhance relative growth rate of *M. aquaticum.*

A high propagule pressure of the invasive alien macrophyte *M. aquaticum* significantly hindered the growth of the native macrophyte community in nutrient-rich conditions, but this effect was not observed in nutrient-poor conditions (**Figures 4A, B**). This finding could imply that the invasive macrophyte may rely on higher nutrient levels to exert its competitive advantage over native macrophyte species, or that native macrophyte species are more resilient or better adapted to nutrient-scarce conditions, which mitigates the impact of invasion by *M. aquaticum*. In high- nutrient conditions, invasive plants often outcompete native species (Gioria and Osborne, 2014; Xue et al., 2023; Shan et al., 2024) due to the invaders' higher growth rates (van Kleunen et al., 2010; Liu et al., 2017) and more efficient resource acquisition and utilization (Funk and Vitousek, 2007). This competitive advantage can lead to the displacement of native species and the formation of invasive plant monocultures (Holdredge and Bertness, 2011). Conversely, in stressful environments with limited nutrients, native plants can outcompete (or at least tolerate) invasive plants as the natives are often better adapted to resource-poor habitats (Funk, 2008; Gioria and Osborne, 2014). Taken together, these findings emphasize that the impact of invasive plant species is not uniform across all habitats and can be influenced by interactive effects of biotic and abiotic factors (Theoharides and Dukes, 2007).

In conclusion, the findings of this study indicate that the invasion dynamics and success of the alien macrophyte *M. aquaticum* are shaped by a combination of factors, including nutrient availability, propagule pressure, competition, and herbivory. These elements influence the growth and competitive ability of the invasive species within the native macrophyte community, underscoring the complexity of invasion processes. Effective management of invasive plants like *M. aquaticum* necessitates a context-specific approach that addresses multiple factors simultaneously, as focusing on a single variable may not adequately control the invasion. For example, when managing *M. aquaticum* invasions, solely focusing on reducing nutrient levels or promoting herbivory might be insufficient if propagule pressure is not simultaneously controlled. Consequently, comprehensive management strategies should concurrently address multiple factors, customized to the specific conditions of each invaded ecosystem.

## Data availability statement

The original contributions presented in the study are included in the article/**Supplementary Material**. Further inquiries can be directed to the corresponding author.

## Author contributions

RH: Writing – original draft. AMOO: Writing – review & editing. YY: Writing – review & editing. WY: Writing – review & editing. CC: Writing – review & editing. LD: Writing – review & editing. SJ: Writing – review & editing. FL: Conceptualization, Writing – original draft.
