# Supplementary material for: Nutrient enrichment, propagule pressure, and herbivory interactively influence the competitive ability of an invasive alien macrophyte Myriophyllum aquaticum
Source: Front Plant Sci. 2024 May 30;15:1411767. doi: 10.3389/fpls.2024.1411767 (PMC11169793; doi:10.3389/fpls.2024.1411767)
Supplement: Supplementary file 1 [file DataSheet_1.docx]

**Table S1** Results of ANOVA that tested main and interactive effects of nutrient availability (N) (low-nutrient vs high-nutrient), propagule pressure (P) (low-propagule vs high-propagule) and herbivory (H) (herbivory vs no-herbivory) and competition by a native macrophyte community (C) (competition vs no-competition) on absolute above-ground biomass of an invasive macrophyte *Myriophyllum aquaticum* . Significant effects (*P* < 0.05) are marked in bold.

| Factor | Above-ground biomass | | | |  |
| --- | --- | --- | --- | --- | --- |
|  |  |  |  |  |  |
|  | df | F value | *P* value |  |  |
| Herbivory (H) | 1 | 29.35 | **0.000** |  |  |
| Nutrient (N) | 1 | 23.24 | **0.000** |  |  |
| Competition (C) | 1 | 13.21 | **0.000** |  |  |
| Propagule pressure (P) | 1 | 231.02 | **0.000** |  |  |
| H*N | 1 | 0.027 | 0.868 |  |  |
| H*C | 1 | 0.089 | 0.765 |  |  |
| N*C | 1 | 1.27 | 0.261 |  |  |
| H*P | 1 | 0.009 | 0.922 |  |  |
| N*P | 1 | 10.49 | **0.006** |  |  |
| C*P | 1 | 1.616 | 0.207 |  |  |
| H*N*C | 1 | 0.004 | 0.947 |  |  |
| H*N*P | 1 | 0.664 | 0.417 |  |  |
| H*C*P | 1 | 0.508 | 0.477 |  |  |
| N*C*P | 1 | 0.015 | 0.902 |  |  |
| H*N*C*P | 1 | 0.456 | 0.501 |  |  |

**Table S2** Results of ANOVA that tested main and interactive effects of nutrient availability (N) (low-nutrient vs high-nutrient), propagule pressure (low-propagule vs high-propagule) and herbivory (H)(herbivory vs no-herbivory) and on proportional above-ground biomass of an invasive macrophyte *Myriophyllum aquaticum*. Significant effects (*P* < 0.05) are marked in bold.

| Factor | Proportional aboveground  biomass | | |  |
| --- | --- | --- | --- | --- |
|  |  |  |  |  |
|  | df | F value | P value |  |
| Herbivory (H) | 1 | 7.94 | **0.007** |  |
| Nutrient (N) | 1 | 5.95 | **0.003** |  |
| Propagule pressure (P) | 1 | 35.64 | **0.000** |  |
| H*N | 1 | 0.060 | 0.806 |  |
| H*P | 1 | 0.007 | 0.931 |  |
| N*P | 1 | 28.19 | **0.000** |  |
| H*N*P | 1 | 0.208 | 0.650 |  |

**Table S3** Results of ANOVA that tested main and interactive effects of nutrient availability (N) (low-nutrient vs high-nutrient), propagule pressure (P) (low-propagule pressure vs high-propagule pressure) and herbivory (H) (herbivory vs no-herbivory) on absolute above-ground biomass of native macrophyte communities. Significant effects (*P* < 0.05) are marked in bold, while marginally significant effects (0.05 ≤ *P* < 0.1) are underlined and in bold.

| Factor | Aboveground biomass | | |  |
| --- | --- | --- | --- | --- |
|  |  |  |  |  |
|  | df | F value | P value |  |
| Herbivory (H) | 1 | 2.19 | 0.083 |  |
| Nutrient (N) | 1 | 8.70 | **0.0051** |  |
| Propagule pressure (P) | 1 | 8.01 | **0.0070** |  |
| H*N | 1 | 0.091 | 0.764 |  |
| H*P | 1 | 0.29 | 0.589 |  |
| N*P | 1 | 2.89 | 0.066 |  |
| H*N*P | 1 | 0.15 | 0.693 |  |


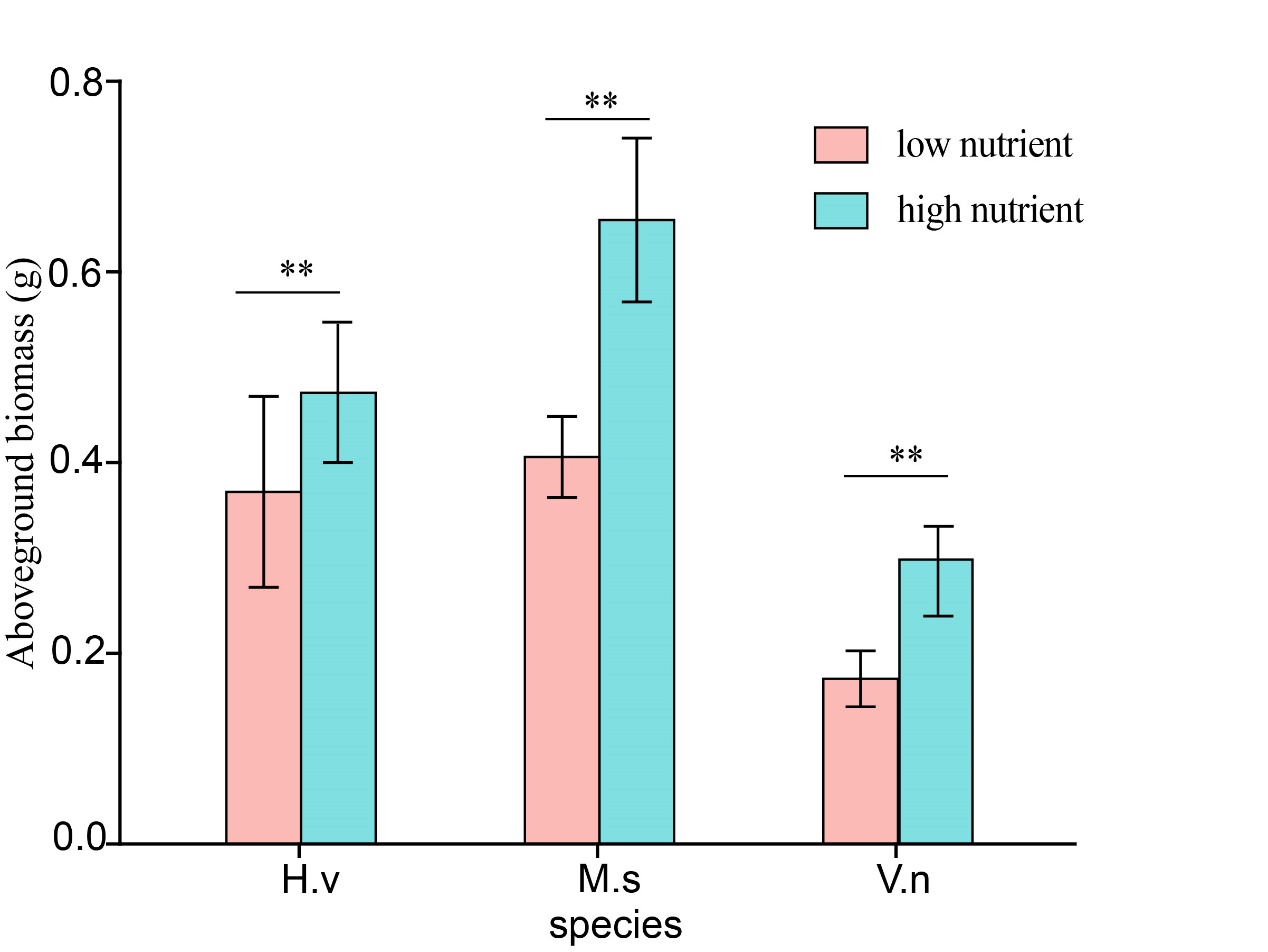


**Figure S1:** Means (±SE) of the three native macrophytes *Vallisneria natans* (H.V), *Hydrilla verticillate* (V.n), and *Myriophyllum spicatum* (M.s) under two levels of nutrient availability (low and high). Symbols above the bars (*: p < 0.05; **: p < 0.01; ns: p > 0.05) indicate statistical differences between low and high nutrient availabilities according to t-test.


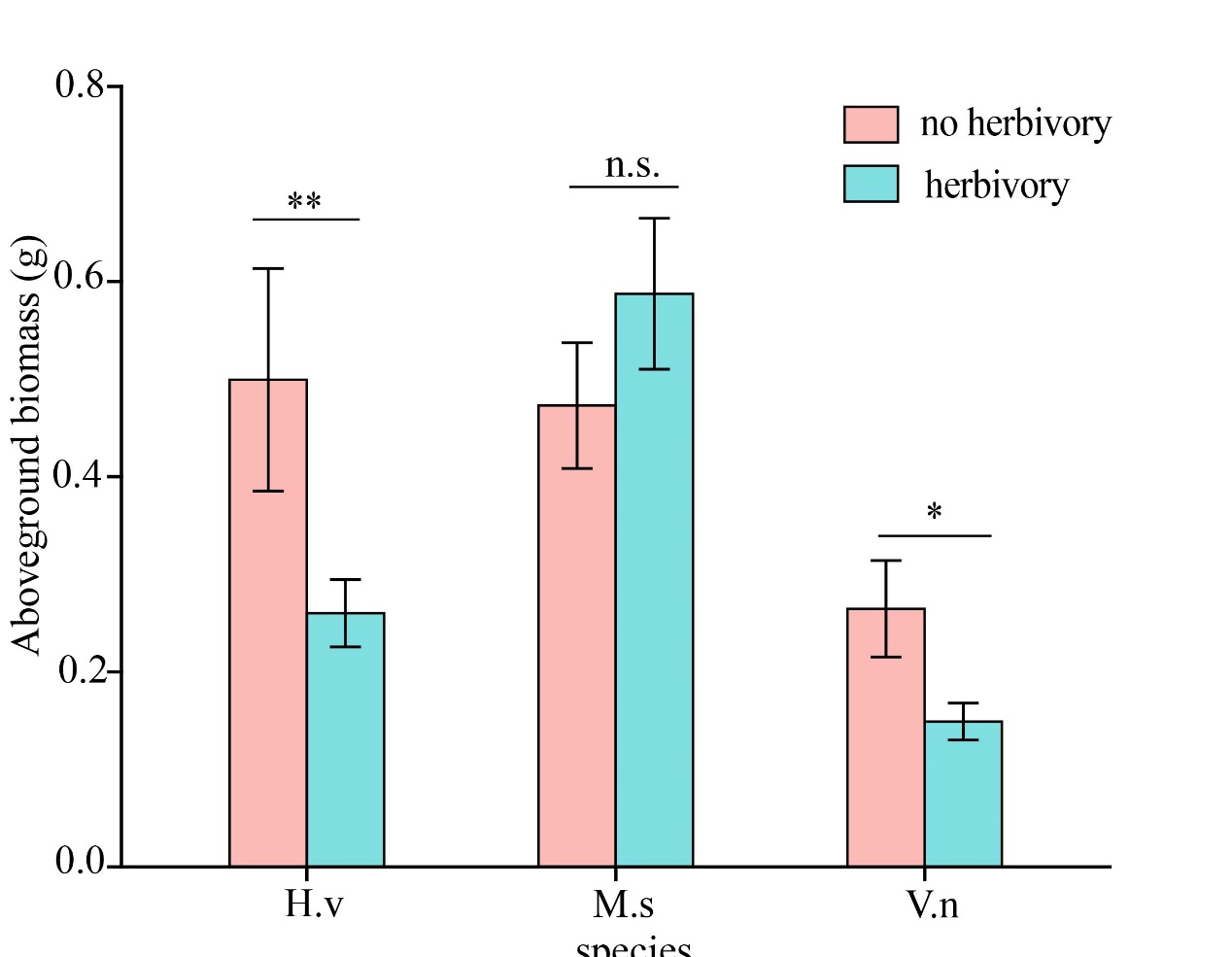


**Figure S2:** Means (±SE) of the three native macrophytes *Vallisneria natans* (H.V), *Hydrilla verticillate* (V.n), and *Myriophyllum spicatum* (M.s) subjected to two herbivory conditions (with and without). Symbols above the bars (*: p < 0.05; **: p < 0.01; ns: p > 0.05) indicate statistical differences between low and high nutrient availabilities according to t-test.
